# Polyphenol-Rich Wild Fruits of the Indian Himalayas as a Potential Nutraceutical Candidate for the Management of Endometriosis: A Review

**DOI:** 10.3390/foods15071178

**Published:** 2026-04-01

**Authors:** Garima Khantwal, Pooja Panthari, Ramesh Kumar Saini

**Affiliations:** School of Health Sciences and Technology, UPES, Dehradun 248007, Uttarakhand, India; garima.khantwal@ddn.upes.ac.in (G.K.); pooja.panthari@ddn.upes.ac.in (P.P.)

**Keywords:** oxidative stress, inflammatory biomarkers, pharmacology, nutraceuticals, *Morus* species, *Berberis asiatica*, *Duchesnea indica*, *Myrica esculenta*, *Rubus ellipticus*

## Abstract

India, home to 4 biodiversity hotspots, hosts 675 wild species used for nutritional and therapeutic purposes. Wild edible fruits are highly valuable for their rich content of health-beneficial compounds, such as polyphenols, carotenoids, and vitamins. The shift in modern lifestyles has increasingly impacted human health. Several factors contribute to heightened oxidative stress, which underpins the development of non-communicable diseases (NCDs). Endometriosis, one of these conditions influenced by oxidative stress, currently lacks a definitive cure, leaving patients reliant on hormonal and surgical treatments. According to the WHO, 10% of girls and women worldwide are affected by endometriosis, often experiencing severe symptoms. This review explores the role of oxidative stress in the progression of endometriosis, its pathophysiology, and the effects of polyphenols found in wild Himalayan fruits, including various phenolic acids, flavonoids, stilbenes, and lignans. It also examines their synergistic effects with other non-polyphenolic compounds in reducing these biomarkers, such as inflammatory enzymes, pro-inflammatory cytokines, and estrogen receptors, and in modulating pathways like NF-κB, PI3K/AKT, among others, based on preclinical and clinical studies. Additionally, the review highlights key wild fruit species native to the Indian Himalayas, details their nutritional and phytochemical profiles, and assesses their potential, individually and synergistically, as functional foods or nutraceuticals for non-invasive treatment options for endometriosis.

## 1. Introduction

Over the past few decades, society has undergone a paradigm shift in socioeconomic development and improved living standards, prompting changes in lifestyle patterns and modern dietary habits. Adopting healthy behaviors, including adequate sleep, regular physical exercise, balanced nutrition, and effective stress management, is considered a way to combat increased oxidative stress and maintain overall health [1,2]. Free radicals are produced in our bodies through processes such as digestion, metabolism, and excretion. If our body is unable to neutralize these free radicals, they can trigger an adverse chain reaction that, to stabilize themselves, can damage the cell membrane, DNA, restrict the action of major enzymes, and disrupt normal cell division [3]. Reactive oxygen species (ROS) are unavoidable byproducts of mitochondrial oxidative metabolism and include hydrogen peroxide, the superoxide anion, and hydroxyl radicals [4].

Oxidative stress has been defined as an imbalance between the production of free radicals, such as ROS, and their neutralisation by antioxidants in the human body. The imbalance is generally seen due to either excessive production of free radicals from endogenous or exogenous sources or a weakened antioxidant system, leading to cellular damage [5]. An increased oxidant environment in the body has been associated with mitochondrial dysfunction, endoplasmic reticulum stress, and activation of NADPH oxidase, which further triggers superoxide production and inflammatory molecule production. A surge in ROS is observed with a high-calorie Western diet, which is mainly composed of fats and carbohydrates [6]. A sedentary lifestyle is a major contributor to global health and a leading risk factor for global mortality, and replacing traditional foods with industrially processed foods poses a disadvantage to the body’s natural defense against free radicals [7]. A risk factor that is strongly associated with increased cardiovascular morbidity and mortality is poor diet quality [8].

Non-communicable diseases (NCDs) constituted around three-quarters of global deaths in 2017, resulting in 41 million deaths annually, primarily driven by lifestyle behaviors, environmental exposure, and genetic predisposition, resulting in action taken in response to reduce one-third of premature mortality by NCDs by 2030 through Sustainable Development Goals [9]. India is facing a growing burden of NCDs, as the India state-level disease burden study by the Indian Council of Medical Research found that 23.9% of deaths increased from 1990 to 2016 were caused by NCDs [10]. A review by Cena et al. [11] defined the role of a healthy diet in combating NCDs, with an emphasis on its definition, which states that a diet rich in macronutrients such as carbohydrates, proteins, and fats, and in micronutrients including vitamins and minerals, should be consumed in balanced proportions to meet the body’s daily physiological requirements and support normal bodily functions. WHO further specified the recommended proportions in which each nutrient should be consumed citing the importance of unrefined carbohydrates representing 45–75% of total daily energy, less than 50 g of free sugars consumption, not more than 10% of saturated fats, 10–15% of protein and appropriate consumption of fruits, vegetables, nuts, legumes, and lean animal source for the intake of micronutrients daily [12]. Research is being conducted to valorize fruits and vegetables and produce polyphenol-rich value-added products. One such review by ‘Aqilah et al. [13] summarized the importance of utilizing the whole fruit pulp, seed, and peel of apple, blackberry, blueberry, strawberry, raspberry, and red dragon fruit, as they are rich sources of bioactive compounds. These parts can be efficiently extracted using solvents and extraction methods to produce juices, wines, yogurts, jams, and nutraceuticals. They can also serve as natural preservatives and indicators in the industry, thereby enhancing food security and health benefits.

**Figure 1:**
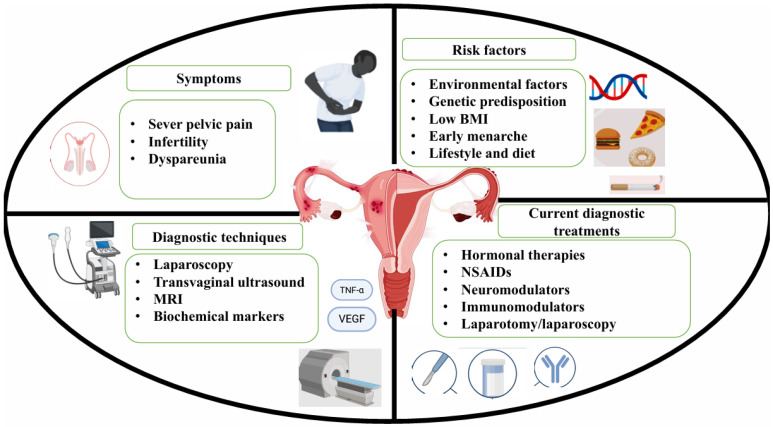
Overview of endometriosis and related factors. Abbreviations: BMI: Body mass index; MRI: Magnetic resonance imaging; NSAIDs: Nonsteroidal Anti-Inflammatory Drugs.

One of the significant physical manifestations associated with oxidative stress that has received comparatively less attention is endometriosis. Endometriosis, initially recognised as an estrogen-dependent localized gynaecological condition, is now considered a complex systemic disease by emerging evidence indicating its effect on metabolic activity in the liver and adipose tissue, promotion of systemic inflammation, and modulation of gene expression in the central nervous system, factors collectively contributing to pain sensitization and neuropsychiatric comorbidities [14,15]. Ectopic endometrial lesions can be distributed throughout the abdominal cavity and are classified as: deep infiltrating endometriosis, superficial peritoneal endometriosis, and ovarian endometriosis, based on anatomical location and depth of invasion [16]. The condition is characterised by symptoms such as pelvic pain, painful menstruation, dyspareunia, and infertility, which also affect other aspects, such as quality of life, by creating a negative impact on social and family life, mental health issues, and substantial healthcare expenses. Standard treatment to mitigate the disease burden and relieve pain symptoms often includes non-steroidal anti-inflammatory drugs (NSAIDs), Gonadotropin-Releasing Hormone (GnRH) agonists, progestins, and oral contraceptives [17]. Given that the disease is hormone-dependent in nature, hormonal or endocrine-based treatments remain primary therapeutic options, which are offered linked to unfavourable mental images and emotions, while causing a broad spectrum of side effects [18]. Surgical treatment is often used to excise or remove ectopic endometriotic tissue. However, consistent improvement is not experienced by all women, considering recurrence remains common, with rates reported as high as 50% within five years [19]. An overview of pathophysiology and current treatment for endometriosis is provided in Figure 1.

Phytochemicals, bioactive compounds derived from plants, play an essential role in disease prevention and treatment due to their diverse pharmacological properties, including antioxidant, anti-inflammatory, and antineoplastic activities [20,21]. Hence, it is essential to understand their causes and the methods for their prevention, using phytochemicals. A review by Osińska et al. [22] concluded that fruits and vegetables may play an important role in the regression of endometriosis, citing the importance of specific foods and diets such as fish oil, green leafy vegetables, and the Mediterranean diet. This review highlighted that these components can modulate the symptoms associated with endometriosis, especially with pain, through the mechanistic regulations of steroid hormones, inflammatory responses, and oxidative stress. In contrast, its opposite, a diet high in processed foods and red meat, has been associated with higher incidences of developing and progression of endometriosis.

The Indian Himalayan region falls within four global biodiversity hotspots, covering 16.2% of India’s total geographical area, spanning about 5.3 lakh km^2^ [23,24]. The Himalayas are divided into three regions: the Greater Himalayas, the Lesser Himalayas, and the Sub-Himalayas. In Uttarakhand, it covers all three zones, with peaks ranging from 8000 m above sea level (asl) to 1000 m asl, and harbours 10,000 plant species unique to the region [25].

This review synthesizes current knowledge on endometriosis, pathophysiology, and pathways, the role of polyphenols, a subclass of phytochemicals, in mediating endometriosis, and in vivo and in vitro studies, with a specific focus on the role of polyphenols from fruits of the Indian Himalayan Region. Furthermore, it will provide evidence on current functional foods on the market, as well as the potential, challenges, and opportunities for functional food and nutraceuticals derived from wild Himalayan fruits.

## 2. Literature Search Methodology

A comprehensive literature search of review articles, research articles, and book chapters was conducted in the electronic databases Google Scholar, PubMed, and Scopus. The primary search keywords used were “endometriosis”, “polyphenols”, “Indian Himalayas”, “north-western Himalayas”, “wild fruits”, “endometriosis treatment”, “phytochemical profiling”, “anti-oxidant activity”, and “inflammatory markers”. Additional search combinations included: (1) endometriosis and prevalence, (2) endometriosis and polyphenols, (3) endometriosis and flavonoids, (4) endometriosis and diet, (5) endometriosis and nutraceuticals. A total of 218 articles published between 2010 and 2025, with particular emphasis on recent publications, were selected and discussed in the review, which focused on Himalayan wild berries, their phytochemical profile, and their antioxidant and anti-inflammatory actions through in vitro, in vivo, and human trials.

## 3. Pathophysiology of Endometriosis

The predominant theory of endometriosis pathogenesis remains Sampson’s theory of retrograde menstruation. According to this theory, viable endometrial tissue refluxes through the fallopian tubes into the peritoneal cavity, where it implants on pelvic organs or peritoneal surfaces [26]. Women who experience early menarche, late menopause, prolonged menstruation, or heavy menstrual bleeding are at an increased risk of endometriosis, further supporting this theory. Another theory, the Müllerian remnants hypothesis, proposes that endometriotic lesions develop in situ via metaplastic transformation or from residual Müllerian duct tissue, which further supports cases of endometriosis in young adolescents shortly after menarche [27]. The lesions are categorised by coloration into white, red, brown, and black, with no relationship to disease severity. The black coloration is attributed to glandular secretions and surrounding stromal reactions [20].

The coelomic metaplasia theory, initially proposed by Iwanoff and Meyer, states that cells from the primitive peritoneum can differentiate into endometrial tissue. The theory stems from the embryological principle that the pelvic peritoneum and ovaries arise from the coelomic epithelium, and it provides evidence for the occurrence of endometriosis in non-menstruating individuals [28]. However, clinical and molecular studies, including recent observations of stromal mutations in endometrium and endometriotic lesions, offer strong evidence that retrograde menstruation is the predominant mechanism responsible for cell deposition in these lesions [29].

The first theory on the origin of stem cells assumed that, during menstruation, epithelial stem cells responsible for the regeneration of the uterine endometrium are shed from the functional layer of the endometrium, may become abnormally activated, and may be confined outside the uterus. Through retrograde menstruation and trans-tubal migration, these adult stem cells may implant in the pelvic cavity, forming ectopic lesions. The second hypothesis regarding the role of stem cells in endometriosis posits that extrauterine stem or progenitor cells, originating outside the uterus, such as bone marrow mesenchymal stem cells or endothelial progenitor cells, may differentiate, leading to the development of ectopic lesions [30].

Redundancy of cellular pathways has been regarded as a key factor in the development of diseases, as it can disguise the genetic and epigenetic alterations that can damage the organisms, along with the fact that multiple pathways have the capability of performing the same biological processes, resulting in mutations that lead to the loss of function, going unnoticed until system failure occurs [31].

Recent studies have examined additional factors that may contribute to the development of endometriotic lesions, including familial tendencies and genetic predisposition. Studies on the pathophysiology of endometriosis have identified several core molecular markers of the disorder, including genetic predisposition, estrogen dependence, progesterone resistance, and chronic inflammation [32]. There is a widely accepted notion that oxidative stress plays a significant role in the pathophysiology of endometriosis, triggering an inflammatory response in the peritoneal cavity [33]. Several inflammatory mediators are released in endometriosis, including prostaglandins, vascular endothelial growth factor (VEGF), tumour necrosis factor-α (TNF-α), nerve growth factor (NGF), and various interleukins (ILs). Cytokines are a group of signalling proteins that can have pro- or anti-inflammatory functions. The group of cytokines includes interferons, interleukins, chemokines, lymphokines, and tumor necrosis factors, and they mediate their effects by binding to specific cell-surface receptors [34]. Cytokines are key molecules in the immune system that play numerous biological roles, including tissue repair, haematopoiesis, and angiogenesis [35].

## 4. Prevalence of Endometriosis

According to the WHO 2023 data, 10% (190 million) of women and girls of reproductive age globally are affected by endometriosis [36]. However, the prevalence of endometriosis in young women is often underestimated due to the neglect of menstrual pain as a symptom, which results in delayed diagnosis, as long as 4 to 11 years after the presentation of the earliest symptoms [31]. Pelvic pain is reported by 50–80% of women with endometriosis, while 50% of affected women have reported infertility. Globally, endometriosis is known to affect 176 million women, but due to the challenging diagnosis condition, 65% of affected women are initially misdiagnosed [37].

## 5. Role of Oxidative Stress in the Progression of Endometriosis

Endometriosis is a well-known disorder characterized by abnormal and uncontrolled growth of endometrial tissue outside the uterus; however, the molecular and cellular mechanisms underlying the condition remain unclear. Pathways involving angiogenesis, cell adhesion and invasion, cell proliferation, apoptosis, and immune system function are supposed to play a role [38]. Endometriosis is predominantly linked to chronic pelvic pain driven by the activation of macrophages and mast cells, which fosters a self-sustaining cycle of inflammation, oxidative stress, and persistent pain. The aim to explore the link between endometriosis and ROS stemmed partly from similarities between the two. However, the former is classified as a benign disorder [39]. Oxidative stress in the local peritoneal environment may play a role in the sequence of events that contribute to the development of endometriosis, which are triggered by factors such as erythrocytes, apoptotic endometrial cells, and residual endometrial cells present in menstrual fluid [33].

The key cause of endometriosis is an altered balance between estrogen and progesterone, as the former promotes the proliferation and maturation of endometrial tissue and the secretion of various proinflammatory cytokines, such as TNF-α, IL-1β, IL-6, and IL-8, by peritoneal macrophages in an estrogen-rich environment [39]. Increased ROS and oxidative stress levels trigger transforming growth factor-β1 (TGF-β1), a cytokine responsible for fibrosis. Once activated, it promotes fibroblast growth and increases the synthesis and accumulation of extracellular matrix components [40]. These components are also known to stimulate the activation and recruitment of mononuclear phagocytes, leading to the generation of lipid peroxides and other byproducts from the interaction of apolipoproteins with peroxides, thereby initiating a localized inflammatory response in the pelvic region [41]. Upregulation of the mitogen-activated protein kinase (MAPK)/extracellular signal-regulated kinase (ERK) pathway is crucial for promoting the proliferation of endometriotic lesions [21].

Studies have shown increased concentrations of IL-1β, IL-6, TNF-α, or high-sensitivity C-reactive protein (hs-CRP) in patients with endometriosis compared to healthy controls [21,42]. Excessive production of ROS activates the NF-kB signalling pathway, leading to upregulation of intercellular adhesion molecule-1 (ICAM-1), resulting in structural changes in peritoneal epithelial cells and the establishment of adhesion sites that support ectopic endometrial attachment [43].

## 6. Key Biomolecular Drivers in Endometriosis

At the cellular level, endometriosis is marked by cell proliferation, persistent inflammation, and excessive angiogenesis, which are interlinked and arise from dysregulated sex hormone signalling, with the estradiol (E2) pathway overactivated and the progesterone (P4) pathway impaired [44].

### 6.1. Cyclooxygenase-2 (COX-2)

Cyclooxygenase is a key rate-limiting enzyme that converts arachidonic acid into prostaglandins. Two COX isoforms are currently known: COX-1, which is present in many tissues, and COX-2, which is induced by mitogens, growth factors, and cytokines. Vascular endothelial growth factor (VEGF), one of the pro-angiogenic cytokines upregulated by COX-2 activity, is markedly overexpressed in several cancers, including ovarian and endometrial cancer, as well as in benign conditions such as endometriosis and spontaneous pregnancy loss [45]. The COX-2/PGE_2_ signalling pathway activates oxygenated fatty acids and is well recognised in a wide range of inflammatory conditions. In endometriosis, this pathway plays a key role in mediating the disease’s associated pain. It is a primary target of non-steroidal anti-inflammatory drugs (NSAIDs). Also, it contributes to the progression of endometriosis by regulating the implantation of ectopic lesions, endometrial cell proliferation, angiogenesis, and immune suppression [46].

### 6.2. Inducible Nitric Oxide Synthase (iNOS)

Nitric oxide is a gaseous free radical that acts as an essential signalling molecule in neurotransmission, vascular regulation, and immune defense, and is produced in the body by nitric oxide synthase via the metabolism of L-arginine to L-citrulline. Nitric oxide synthase enzymes are distributed in various organs and cell types, including macrophages, endothelial and neuronal cells, fibroblasts, platelets, and hepatocytes. They are categorised by origin and functional properties into neuronal NOS, inducible NOS, and endothelial NOS [47].

Several studies have suggested an association between elevated nitric oxide levels and the progression of endometriosis. iNOS expression is widely reported to be upregulated in affected women, with ectopic lesions exhibiting substantially greater NO production [48,49].

### 6.3. Pro-Inflammatory Cytokines

Macrophages play an essential role in recognizing foreign cells, which are more abundant in the peritoneal cavity of women with endometriosis. The cells release increased levels of pro-inflammatory cytokines. In contrast, anti-inflammatory signalling from stromal, epithelial, smooth muscle, and other immune cells is reduced, favouring the microenvironment for the initiation and progression of the disease [50].

Research indicates that high levels of inflammatory cytokines, including IL-6, IL-8, TNF-α, and VEGF, are present in endometriomas [51]. Trapped blood within the cysts leads to iron accumulation, which, in turn, drives the production of large amounts of ROS and increases inflammation. Follicular fluids have also been reported to contain elevated levels of inflammatory cytokines and higher ROS, which directly interfere with follicle growth and damage the oocyte [52,53].

Interferon-γ (IFN-γ) plays a key role in modulating the Th1/Th2 balance and has been reported to show altered expression in endometriosis studies [48]. Although its levels are reduced in peripheral blood, IFN-γ remains elevated in peritoneal fluid and ectopic lesions, and ectopic tissues exhibit higher IFN-γ mRNA expression, indicating localized upregulation at lesion sites [54]. A study of 59 women with confirmed endometriosis assessed IL-6 as a diagnostic marker using a machine-learning model, given that IL-6 levels are elevated in these women. This study opens the path for further non-invasive and quick ways to diagnose endometriosis based on levels of inflammatory markers [55].

### 6.4. Nuclear Factor-κB (NF-κB) Pathway

NF-κB, a central regulator of immune and inflammatory responses, governs proliferation, adhesion, apoptosis, invasion, and angiogenesis, thereby linking early development and progression of endometriotic lesions. The activation of the NF-κB pathway by factors such as IL-1β, Tumor Necrosis Factor (TNF)-α, and Toll-like receptor (TLR)4 contributes to both the initiation and progression of endometriosis. In the early stages, NF-κB upregulates key adhesion molecules, including DcR3, CD44, ICAM-1, and VCAM-1, supporting the abnormal attachment of endometrial cells to ectopic sites. As disease progression advances, NF-κB activity promotes the production of cytokines and chemokines, such as IL-1β, IL-6, IL-8, TNF-α, IFN-γ, eotaxin, and RANTES, as well as proliferative markers, such as Proliferating Cell Nuclear Antigen (PCNA), thereby maintaining a chronic inflammatory state [56,57]. An in vitro study on primary endometrial stromal cells showed that aspirin and aloe-emodin treatment successfully inhibited NF-κB signalling by downregulating IKK activity, thereby reducing proliferative capacity and downregulating adhesion molecules such as ICAM and VCAM, highlighting NF-κB as a central therapeutic target [58].

### 6.5. Matrix Metalloproteinases (MMPs)

Matrix metalloproteinases are a family of zinc-dependent endopeptidases, including collagenases, gelatinases, and stromelysins, that degrade and remodel the extracellular matrix. Their activity is precisely controlled by tissue inhibitors of metalloproteinases, which help regulate matrix dynamics in both healthy and diseased conditions. In endometriosis, these enzymes play a crucial role in endometrial tissue remodeling throughout the menstrual cycle. A study involving 43 patients with visible stage 2 and 3 peritoneal endometriotic lesions found increased levels of MMP-2 and MMP-9 in the endometrium of patients with endometriosis compared to controls. This suggests a strong link between MMP expression in ectopic and eutopic endometrial tissue and indicates their potential as non-invasive diagnostic markers for endometriosis [59].

Another role MMPs play in the progression of endometriosis is in angiogenesis and vasculogenesis, which establish and maintain ectopic endometrial lesions by degrading the extracellular matrix and promoting endothelial cell migration. Studies have indicated that MMP-1 promotes Vascular endothelial growth factor receptor (VEGFR)-2 expression, while MMP-7 enhances VEGF signalling by degrading VEGFR-1, thereby creating a supportive environment for angiogenesis. Further, MMPs are known to contribute to immune evasion by cleaving natural killer group 2 member D (NKG2D) ligands and ICAM-1, thereby impairing NK-mediated cytotoxicity [60].

### 6.6. Transforming Growth Factor β (TGF-β)

The TGF-β superfamily comprises a group of cytokines that is involved in the pathophysiology of endometriosis. It promotes prolactin production and triggers decidual-like differentiation in ectopic endometrial stromal cells (ESCs) [61]. TGF-β occurs in three isoforms, i.e., TGF-β 1, TGF-β 2, and TGF-β 3, and is considered essential for endometrial function; however, upregulation of TGF-β 1 promotes disease progression by inducing epithelial–mesenchymal transition, increasing MMP expression, inhibiting apoptosis, promoting angiogenesis, and suppressing the immune response [62].

### 6.7. VEGF (Vascular Endothelial Growth Factor)

Survival of ectopic endometrial tissue in the peritoneal cavity depends on the formation of a dense vascular network that provides adequate oxygen and nutrient support. Neovascularisation in endometriotic lesions is achieved through two primary mechanisms: vasculogenesis and sprouting angiogenesis. Vascular endothelial growth factor and fibroblast growth factor-2 (FGF-2) are primary mediators of endothelial progenitor cell migration and proliferation. VEGF, a potent angiogenic mediator, is upregulated by estradiol in endometrial cells during the late proliferative phase of the menstrual cycle. VEGF is overexpressed in endometriotic lesions, supporting its involvement in lesion vascularisation [63].

### 6.8. Estrogen Receptors

Estrogen plays a crucial role in female reproduction through two estrogen receptors, ERα and Erβ. In the follicular phase of the menstrual cycle, estrogen, via its receptors, stimulates endometrial growth, leading to thickening of the endometrial mucosa. A key biomarker involved in the development and persistence of endometriotic tissue is 17β-Estradiol (E2), which is well associated with inflammation and pain. Estradiol is predominantly produced locally within the lesions; it can also reach endometriotic lesions via the circulation. The local accumulation of estrogen plays an important role in the development and progression of endometriosis by binding to and activating estrogen receptors. This process is thought to be caused by altered activities of enzymes responsible for estradiol biosynthesis and inactivation [64,65].

**Figure 2:**
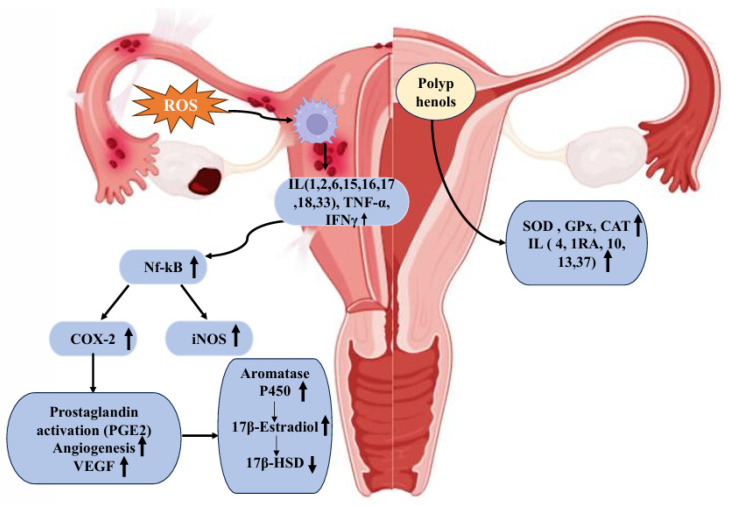
Biomarkers involved and their interactions in the promotion of endometriosis. Abbreviations: ROS: reactive oxygen species; IL: interleukins; TNF-α: Tumor necrosis factor-alpha; IFN-γ: interferon-gamma; NF-κB: nuclear factor kappa-light-chain-enhancer of activated B cells; COX-2: cyclooxygenase-2; iNOS: inducible nitric oxide synthase; VEGF: vascular endothelial growth factor; Aromatase P450: aromatase cytochrome P450; 17β-HSD: 17-beta-hydroxysteroid dehydrogenase; SOD: superoxide dismutase; GPx: glutathione peroxidase; CAT: catalase. ↑ indicates upregulation and ↓ indicates downregulation.

In normal endometrium conditions, Erα is expressed at higher levels than Erβ and is mainly associated with cell proliferation. In endometriosis, reduced Erα expression is observed, accompanied by marked upregulation of Erβ [66]. An in vitro study conducted with endometrial stromal cells obtained from women with confirmed endometriosis showed that Erβ regulated CCL2 (C-C motif chemokine ligand 2) production through NF-κB signalling in endometrial stromal cells, further promoting macrophage recruitment to ectopic lesions and contributing to the pathogenesis of endometriosis [67]. The effects of these biomarkers on endometriosis progression are shown in Figure 2.

## 7. Most Researched Polyphenols in Mediating Symptoms of Endometriosis

Polyphenols are plant secondary metabolites with over 8000 identified structures. Their biological activities primarily stem from their capacity to neutralize ROS and reactive nitrogen species (RNS) by donating electrons, thereby lowering oxidative stress and inflammation [68,69]. Polyphenols are classified by their chemical structure as follows: (1) phenolic acids, which contain a single phenolic ring; (2) flavonoids, featuring two aromatic rings connected by a three-carbon chain that includes one oxygen atom; (3) stilbenes, composed of two aromatic rings linked by a methylene bridge; and (4) lignans, characterized by a diphenolic structure where two phenylpropane units are joined by a carbon-carbon bond [70]. A systematic review paper by Tassinari et al. [71] highlighted the role of diet in advancing or reducing endometriosis, noting that certain animal and plant-derived compounds, such as arachidonic acid (an omega-6 polyunsaturated fatty acid), can promote inflammation. The authors also highlighted the importance of flavonols, flavones, isoflavones, and stilbenoids in influencing key molecular pathways, such as the phosphoinositide 3-kinase (PI3K)/protein kinase B (AKT)/mammalian target of rapamycin (mTOR) and NF-κB pathways, which are crucial to the pathophysiology of endometriosis, as mentioned earlier. The authors mentioned that studies have reported that these compounds suppress COX-2 expression, reduce pro-inflammatory mediators, and prevent the overproduction of prostaglandins. This section will further explore additional research on other polyphenolic compounds.

### 7.1. Baicalein

**Figure 3:**
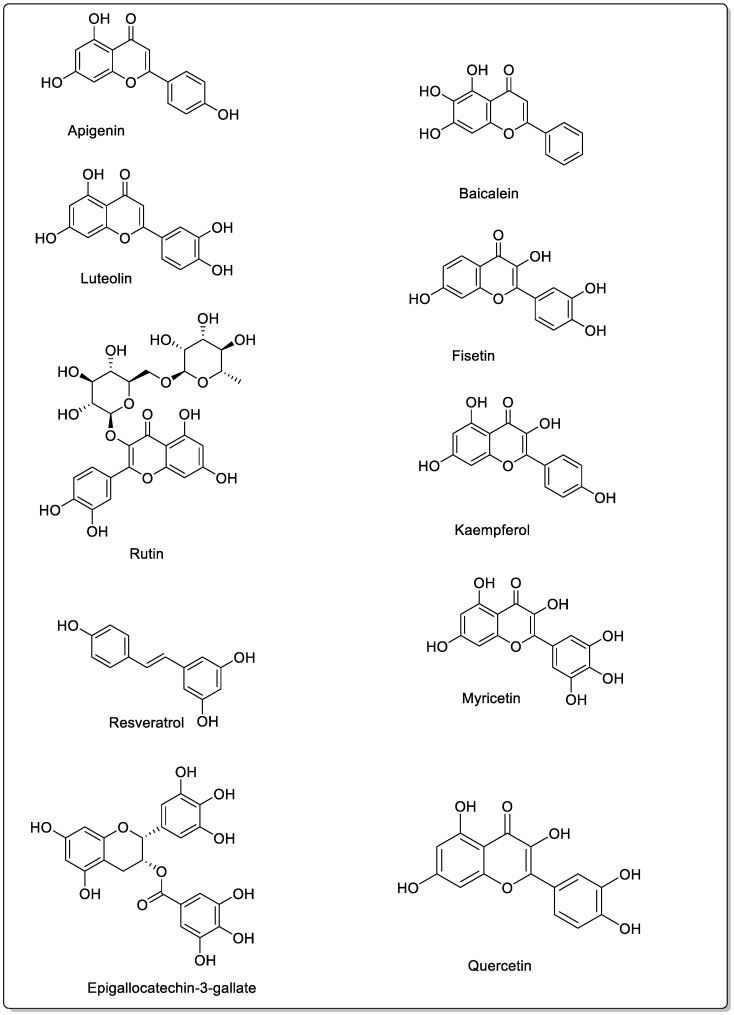
Chemical structures of the most researched polyphenols.

Baicalin and baicalein are two bioactive flavonoids known for their broad pharmacological activities, including anti-inflammatory, antioxidant, antiviral, and anticancer effects. Baicalin exhibits poor oral bioavailability, primarily due to its low aqueous solubility and limited membrane permeability. In contrast, its aglycone metabolite, baicalein, shows greater lipophilicity and enhanced intestinal permeability following removal of the glucuronic acid moiety. The hydrolysis of baicalin to baicalein occurs in the intestine by gut microbiota and enzymes, thereby increasing its bioavailability [72]. The presence of a di-Orthohydroxyl group on ring A is the key feature of baicalein [73] (Figure 3).

A study conducted by Ke et al. [74] to examine the effect of baicalein on the invasion of ectopic endometrial stromal cells in vitro and in vivo revealed significant dose-dependent suppression of the invasive capacity of ectopic endometrial stromal cells treated with increasing concentrations of baicalein (0–40 μmol/L) for 48 h. Wound-healing assay results were consistent with the findings, revealing that baicalein also effectively suppressed the migration of ectopic endometrial stromal cells. Downregulation of TGFB1 expression was also observed in baicalein–treated mice.

### 7.2. Fisetin

Fisetin is a naturally occurring flavonoid with a well-defined chemical structure, widely found in fruits and vegetables, particularly in cucumber, strawberry, and apple, with content ranging from 0.1 to 160 μg/g [75]. It exhibits a broad spectrum of pharmacological activities, including anti-inflammatory, antioxidant, neuroprotective, and anticancer properties, which suppress tumour growth, angiogenesis, invasion, migration, and autophagy, highlighting its potential as a multifunctional bioactive compound in disease prevention and therapy [76,77]. Fisetin is characterised by a diphenylpropane framework, consisting of two aromatic rings linked through an oxygenated heterocyclic ring (Figure 3). The biological activity of fisetin is governed particularly by hydroxyl groups at the 3, 7, 3′, and 4′ positions, the carbonyl group at C4, and the double bond between C2 and C3 [78].

Arangia et al. [79] reported that the antioxidant effects of fisetin in Sprague-Dawley rats are modulated through the NLRP3 (NOD-like receptor family pyrin domain-containing 3 inflammasome) pathway. In this study, fisetin treatment significantly reduced cyst size, histological alterations, mast cell activity, fibrosis markers, and inflammatory cell infiltration.

### 7.3. Quercetin

A naturally occurring flavanol widely found in flowers, barks, stems, roots, wines, teas, and a variety of vegetables and fruits, commonly apples, berries, and onions. Composed of a flavonoid skeleton, it contains three benzene rings and five hydroxyl groups (Figure 3). It exists in the aglycone form, lacking a carbohydrate moiety. It is biosynthesised through the phenylpropanoid pathway and involves key enzymes such as cinnamate 4-hydroxylase, phenylalanine ammonia-lyase, p-coumarate: CoA ligase (4-CL), chalcone isomerase (CHI), chalcone synthase (CHS), flavanone 3β-hydroxylase (F3H), flavonol synthase (FLS), and flavonol 3′-hydroxylase (F3′H) [80,81]. Interestingly, quercetin accounts for nearly 75% of that daily intake of flavonoids [82].

A study conducted by Delenko et al. [83] observed a dose-dependent inhibitory effect of quercetin on the proliferation of menstrual effluent-derived endometrial stroma cells isolated from and cultured from healthy women and women with histologically confirmed endometriosis of menstruating age, mimicking cAMP and MPA. In this study, quercetin reduced the proliferation of menstrual effluent-derived endometrial stroma cells obtained from healthy controls in a dose-dependent manner (6.25–50 µM). Moreover, in cells obtained from patients with endometriosis, a significant inhibition was observed at 25 µM.

### 7.4. Kaempferol

Kaempferol, a flavonol also referred to as triterahydroxyflavone, is mainly found in plants as glycosides, including astragalin, populnin, nicotiflorin, and kaempferitrin. Kaempferol has a diphenylpropane backbone and is biosynthesized via the phenylpropanoid pathway [84,85] (Figure 3).

In a study by Chuwa et al. [86], Kaempferol inhibited the proliferation of the human endometrial adenocarcinoma cell lines Ishikawa (estrogen receptor-positive) and HEC 265, with IC50 values of 83 µM and 65 µM, respectively. Apoptosis suppression was observed through downregulation of estrogen receptor α and the anti-apoptotic proteins survivin and Bcl-2, accompanied by p53 activation and PARP cleavage.

### 7.5. Myricetin

Isolated from the bark of *Myrica rubra*, myricetin is a polyhydroxyflavonol widely distributed across plant families, including Vitaceae, Myricaceae, Leguminosae, Ericaceae, Rosaceae, Compositae, and Fagaceae. Humans commonly consume myricetin through berries, fruits, vegetables, honey, red wine, and tea. Although its stability is temperature-dependent, studies have indicated that myricetin remains stable under strong acidic conditions and occurs in both free form and various glycosides, including arabinosides, rhamnosides, galactosides, xylosides, and galloylated derivatives [87,88,89] (Figure 3).

Park et al. [90] investigated the role of myricetin in endometriosis in female mice and in VK2/E6E7 (VK2) and End1/E6E7 (End1) patient-derived endometriosis cell lines. In this study, a dose-dependent (0–100 μM) inhibitory trend was observed in the VK2 and End1 cell lines, resulting from cell cycle arrest in the sub-G0/G1 phase. Mitochondrial membrane depolarization was observed in the VK2 and End1 cell lines, and an elevation in cytosolic calcium was observed at 100 μM myricetin. The study also indicated that myricetin inhibited cervical cell growth by suppressing PI3K/AKT and MAPK/ERK survival signalling while activating the pro-apoptotic p38 pathway. The combination of myricetin with pathway-specific inhibitors (LY294002, U0126, SB203580) enhanced antiproliferative activity, particularly in VK2 cells. Moreover, a reduction in the size of endometriotic lesions was observed in mice treated with 30 mg/kg/day myricetin for 4 weeks, as compared to DMSO-injected mice, thus demonstrating its potential as a therapeutic agent.

### 7.6. Epigallocatechin-3-Gallate

Epigallocatechin-3-gallate (EGCG) consists of three aromatic rings connected by a pyran ring (Figure 3). It is primarily absorbed in the intestine, where the gut microbiota plays a key role in its metabolism by converting large polyphenolic structures into smaller bioactive metabolites, thereby enhancing its systemic efficacy. Its pharmacological activity is primarily attributed to its multiple phenolic hydroxyl groups, which also make it important in the food industry. The primary source of EGCG is brewed green tea, and it is associated with protective effects against various inflammatory diseases and cancers [91,92,93]. Activation of multiple signaling pathways involved in inflammation and carcinogenesis, including activator protein-1, NF-κB, signal transducer and activator of transcription-3, MAPK, intercellular adhesion molecule, and COX-2, results from the shift toward oxidative stress, which can be modulated by EGCG [94].

A study by Laschke et al. [95] examined the anticancer properties of epigallocatechin-3-gallate and its effectiveness against endometriosis in cultured endometrial glandular and stromal cells from the uterus of 10 hamsters, and in an in vivo model of hamsters. The study concluded that the green tea polyphenol epigallocatechin-3-gallate suppressed cell proliferation, estradiol-induced activation, and VEGF expression in endometrial cells, decreased angiogenesis and blood flow in ectopic endometrial tissue, and promoted regression of endometriotic lesions. Another study by Di et al. [96] demonstrated the effect of epigallocatechin-3-gallate on lipopolysaccharide (LPS)-induced bovine endometrial epithelial cells in female mice. LPS strongly enhanced TNF-α, IL-1β, and IL-6 levels. At the same time, epigallocatechin-3-gallate significantly downregulated their expression, indicating its role in inflammatory and oxidative stress, as well as in deregulating apoptosis in uterine tissue.

### 7.7. Apigenin

Apigenin is a widely distributed plant flavonoid belonging to the flavone subclass, predominantly found in Asteraceae, Lamiaceae, and Fabaceae. In plants, apigenin occurs as the aglycone and in various conjugated forms, including C- and O-glucosides, O-methyl ethers, glucuronides, and acetylated derivatives (Figure 3). It is biosynthesized mainly through the phenylpropanoid pathway from shikimate-derived amino acids phenylalanine and tyrosine. Apigenin is notable for inhibiting the cytochrome P450 enzyme CYP2C9, which plays a key role in the oxidation of drugs and xenobiotics, underscoring its importance in pharmaceutical research [97,98]. Apigenin exhibits low water solubility and pronounced lipophilicity, facilitating efficient membrane penetration [99].

In a study by Liang et al. [100], the inhibitory effects of apigenin on cell viability, migration, invasion, and apoptosis induction in Ishikawa EC cells were investigated. Results showed a progressive reduction in colony formation in Ishikawa cells as apigenin concentrations increased from 20 to 70 μM, indicating a concentration-dependent inhibitory effect. Untreated cells showed significantly greater migration into the wound area than apigenin-treated cells after 48 h. The Transwell assay revealed that API treatment suppressed the migration and invasion abilities of human endometrial carcinoma Ishikawa cells.

### 7.8. Luteolin

A bioactive flavonoid metabolite is well recognized for its potent antioxidant and anti-inflammatory properties, as evidenced by its long-standing traditional use in Chinese medicine to treat a wide range of disorders [101]. Luteolin has four hydroxyl groups on its flavone skeleton. It is sensitive to ambient oxygen, which both contributes to its antioxidant function and influences its stability (Figure 3). Studies have shown that luteolin has high intestinal absorption and is classified as a human intestinal absorption-positive compound [102,103]. The biosynthesis of luteolin in plants begins with the amino acid phenylalanine, which is converted to trans-cinnamic acid by phenylalanine ammonia-lyase. Luteolin can also be obtained by the conversion of naringenin via flavone synthase [104].

A study by Park et al. [105] showed that luteolin inhibited cell proliferation in the VK2/E6E7 and End1/E6E7 cell lines, with 44% and 43% reductions at 20 μM after 48 h, respectively. The researchers used these concentrations for subsequent experiments, in which significant decreases in PCNA expression and cell-cycle arrest in the sub-G0/G1 phase were observed, likely mediated by downregulation of MAPK and PI3K/AKT signaling, as well as suppression of CCNE1 (Cyclin E1) mRNA. A significant reduction in the size of endometriotic lesions was observed in mice treated with 40 mg/kg of luteolin by ip injection.

### 7.9. Resveratrol

Resveratrol, a representative of hydroxy stilbenes, which are a class of naturally occurring polyphenolic compounds that are extensively studied for their cytoprotective potential due to their potent antioxidant activity through free radical scavenging [106]. Structurally, resveratrol has two phenolic rings linked by a styrene double bond and exists as cis and trans isomers, of which the latter is more stable, more biologically active, and predominantly found in nature [107] (Figure 3).

Resveratrol is commonly present in dietary sources as the glycosylated form piceid. Due to this glycosylation, resveratrol is protected from enzymatic degradation, thereby enhancing its stability, biological activity, and overall bioavailability [108]. Initially, it was identified as an immunomodulatory agent for its ability to suppress the proliferation of splenic lymphocytes induced by concanavalin A, interleukin-2, and alloantigens, and to exert a greater inhibitory effect on lymphocyte production of IL-2 and IFN-γ, as well as macrophage secretion of TNF-α and IL-12 [109].

In a study by Gołąbek et al. [110], the therapeutic potential of resveratrol and its natural analogs was investigated on a macrophage-endometriotic cell co-culture model, focusing on their effects on inflammatory and oxidative stress markers. Overall, downregulation of pro-inflammatory genes and reduced ROS production were observed, suggesting that stilbenes can modulate the immune microenvironment and ameliorate oxidative stress. In another study by Chen et al. [111] investigating the role of resveratrol in endometriosis, two models were employed: one in rats and the other in human ectopic endometrial stromal cells. The results show a significant reduction in lesion size and improvement in lipid profiles in rats. In vitro, resveratrol reduced cell proliferation and invasion while promoting apoptosis. Another study by Arablou et al. [112] on endometrial stromal cells found that resveratrol at 100 µM significantly decreased gene and protein expression of TGF-β, VEGF, and MMP-9 in ectopic and eutopic endometrial stromal cells at different time points.

### 7.10. Rutin

Rutin, also called rutoside, quercetin-3-O-rutinoside, or vitamin P, is a natural flavonol glycoside made of quercetin attached to the disaccharide rutinose (Figure 3) and is commonly present in foods such as asparagus, buckwheat, apricots, apples, cherries, grapes, grapefruits, plums, citrus fruits, and tea [113]. Rutin has been known for various pharmacological activities, including antioxidant and anti-inflammatory [114], anticancer [115], and neuroprotective [116], among others [117].

In a study by Talebi et al. [118] on female Wistar albino rats, in which endometriosis was induced surgically, rutin at 3000 and 6000 µg/kg upregulated Bax and cleaved caspase and downregulated Bcl-2, confirming the rutin-mediated induction of apoptosis. Additionally, concentrations of SOD, GPx, and total antioxidant activity (TAC) were higher in treated animals, suggesting greater antioxidant potential. In another study by Chen et al. [119] on ZEA-induced apoptosis in porcine endometrial stromal cells, rutin at 25 μM enhanced SOD and GSH-Px activity, reduced ROS and peroxide accumulation, upregulated MMP, and decreased the Bax/Bcl-2 ratio through the Nrf2 pathway.

### 7.11. Anthocyanins

Among flavonoid categories, the water-soluble pigments, anthocyanins, are predominantly found in plant sources, often in glycosylated forms. They have a significant role in imparting distinctive red, blue, and purple colours to various plant tissues [120]. Investigations have highlighted the role of dietary anthocyanins and their numerous health-promoting effects, including mitigating neurological and cardiovascular disorders, various types of cancer, and, due to their anti-inflammatory properties, the management of various chronic conditions [121].

Jiang et al. [122] investigated the effects of anthocyanin-enriched preparations from mulberry juice and mulberry marc purification in mice using a weight-loaded swimming test, which demonstrated enhanced endurance compared to the control group, further supporting the hypothesis that anthocyanins mitigate oxidative stress induced by strenuous exercise through their antioxidant capabilities.

Park et al. [123] investigated the antiproliferative and pro-apoptotic effects of delphinidin, derived from eggplants, berries, and red onions, in VK2/E6E7 and End1/E6E7 cell lines derived from vaginal and endocervical tissues of premenopausal women. The findings indicated that delphinidin, administered in a dose-dependent manner, significantly inhibited cell proliferation, with a 100 μM dose reducing proliferation by about 50% compared to vehicle-treated controls and decreasing PCNA expression. Additionally, delphinidin decreased mitochondrial membrane potential and cytosolic calcium levels in a dose-dependent manner and suppressed the PI3K/AKT signalling pathway.

In addition to the major polyphenols discussed above, several other non-polyphenolic compounds have been identified and studied for their effects on endometriosis. These compounds can be found in the wild fruits of the Indian Himalayas, discussed in the upcoming sections. A molecular docking study by Liu et al. [124] investigated a traditional Chinese medicine, Wenjing decoction, used to treat endometriosis. They identified and filtered 50 compounds from 8 herbs, with the top five bioactive compounds being quercetin, kaempferol, wogonin, β-sitosterol, and stigmasterol, which exhibited the highest binding affinity with the top four targeted genes responsible for endometriosis. These findings highlight the potential synergistic interactions between polyphenolic and non-polyphenolic compounds to modulate molecular pathways involved in endometriosis. Hence, keeping this in mind, several other polyphenolic and non-polyphenolic compounds that synergize to mediate endometriosis symptoms are summarized in Table 1.

## 8. Clinical Trials on Polyphenols in Mediating Symptoms of Endometriosis

Clinical trials are very limited in endometriosis research and have primarily focused on hormonal pathways, targeting estrogen and progesterone receptors, as well as gonadotropin-releasing hormone analogs, to assess an incompletely defined mechanism, reflecting the complexity of the disease. While hormonal therapies dominate the therapeutic landscape, there is an emerging trend toward non-hormonal approaches and natural-based interventions [137]. Maia Jr et al. [138] conducted an open-label study to assess the effect of resveratrol (30 mg/day) in combination with an oral contraceptive containing drospirenone (3 mg) and ethinylestradiol (30 μg) in 12 reproductive-age patients. The patients reported a decrease in pain; however, the symptom persisted. In the second group of the same study, 42 patients received the same combination to assess its effect on COX-2; COX-2 levels were significantly reduced, suggesting that combining natural compounds with oral contraceptives is an effective treatment strategy compared with oral contraceptives alone.

Signorile et al. [139] conducted a prospective case–control study where ninety patients were selected to examine the effect of a combination of natural ingredients containing linoleic acid (1002 mg), α-linolenic acid (432 mg), quercetin (200 mg), nicotinamide (20 mg), 5-methyltetrahydrofolate calcium salt (400 µg), titrated turmeric (20 mg), and titrated parthenium (19.5 mg) on inflammatory response and symptoms in endometriosis. The study revealed a significant reduction in symptoms among patients treated with the combination, including headache, cystitis, muscle ache, irritable bowel, dysmenorrhea, dyspareunia, and chronic pelvic pain. Furthermore, serum levels of prostaglandin E2, 17β-estradiol, and CA-125 decreased markedly. Another study by Rostami et al. [140] assessed the effects of an astaxanthin supplement (6 mg) on antioxidant, anti-inflammatory, and fertility-enhancing activities over 12 weeks in a randomized, triple-blind, placebo-controlled trial involving 50 infertile patients with endometriosis. The study found higher serum levels of total anthocyanins and SOD. The supplementation group showed downregulation of MDA, FF, TNF-α, and IL-6 compared with the placebo group.

A recent meta-analysis by Meneghetti et al. [141] assessed 11 randomized clinical trials, including 716 women, that evaluated the effects of dietary interventions, including antioxidants and phenolic compounds, on alleviating endometriosis symptoms. Of these, 6 compared a specific antioxidant combination with a placebo group. The meta-analysis consistently reported reductions in symptom dysmenorrhea, but not in dyspareunia or chronic pelvic pain, suggesting that bioactive compounds may directly and effectively downregulate pro-inflammatory cytokines and inhibit PEG2 and COX-2, ultimately affecting inflammatory pathways. Supplementation included vitamin D, pycnogenol, vitamin C, vitamin E, garlic, palmitoylethanolamine, transpolidatin, Wobenzym Vital, minerals (Ca, Mg, Se, Zn, Fe), probiotics, and fish oil.

Barrea et al. [142] reviewed studies on the effects of Mediterranean, ketogenic, and nutrient-supplemented diets on endometriosis symptoms and summarized that, due to its richness in antioxidant and anti-inflammatory compounds such as vitamins, minerals, phenolic compounds, and omega-3 PUFAs, the Mediterranean diet can help to minimize pelvic pain and endometriosis. The Mediterranean diet primarily comprises fruits, vegetables, legumes, whole grains, nuts, fish, and extra virgin olive oil, with minimal or no consumption of red and processed meats and refined sugar products. The review also synthesised findings on the most commonly used and researched nutritional supplements, such as vitamin D, C, and E, curcumin, and quercetin, in which most clinical trials reported reductions in symptoms, including dysmenorrhea and pelvic pain.

## 9. Overview of Biodiversity of Wild Fruits of the North Western Himalayas

The Indian Himalayan region spans 26°20′–35°40′ N latitude and 74°50′–95°40′ E longitude, covering the Himalaya and adjoining hills of Himachal Pradesh, Jammu and Kashmir, Uttarakhand, Sikkim, Meghalaya, Nagaland, Arunachal Pradesh, Manipur, and Mizoram, along with hill districts of Assam and West Bengal [143]. A study conducted by Aryal et al. [144] in a part of the Kailash Sacred Landscape, reported that families of wild and non-cultivated edible plants, namely Moraceae, Rosaceae, Urticaceae, Polygonaceae, Araceae, Dioscoreaceae, Amaranthaceae, Lamiaceae, and Combretaceae, were found and utilized, most commonly for food, medicinal, and spice uses, and other purposes, highlighting the importance of biodiversity of the region. Uttarakhand, being an integral part of the Indian Himalayan Region, is located between the latitudes 28°44′ and 31°28′ N and longitudes 77°35′ and 81°01′ E. The state is split into two regions, Garhwal and Kumaon, with 13 districts in total, covering an area of 53,483 km^2^, most of which is rugged terrain, steep slopes, and snow-covered regions [145]. Uttarakhand’s biodiversity is especially notable because the region lies within internationally recognized biodiversity hotspots, underscoring its ecological importance [146].

## 10. Importance of Utilising Underutilized Plants in Traditional Medicine and Nutrition

The Terai region of Uttarakhand is a flat stretch in the south of the Himalayan foothills, composed of fine alluvium and clay-rich swamps, and is marked by high precipitation and humid conditions, which favour soils rich in minerals and organic matter. Traditionally, many underutilised fruits from this region have been used in folk medicine to treat conditions such as fever, cough, cold, and skin conditions. Many of these wild fruits from the regions are seldom eaten or processed due to their seasonal availability and short shelf life, and thus are underutilized, despite their nutritional value, which includes an abundance of carbohydrates, vitamins, minerals, and bioactive compounds, making them a strong potential source for application in health-promoting initiatives [147,148]. Various fruits and their traditional uses found in these regions highlight the historical therapeutic relevance of these plants and support their potential as sources of bioactive compounds with antioxidant and anti-inflammatory properties, as mentioned in Table 2.

The central Himalayan region of Uttarakhand comprises a large variety of wild edible plants (338 species). The inhabitants of mountain communities possess extensive indigenous and traditional knowledge regarding the sustainable harvesting and utilization of these wild resources. However, the true potential of these wild edibles remains underutilized and underdocumented [160]. Several value added products from these fruits can be seen in market seasonally such as wine, vinegar, jam, jellies, squashes and ready to serve drinks by local people of the region but does not gain much attention and profit due to short shelf life as well as commercial scale production despite natural antioxidants gaining significant importance for their safety and diverse application in food, pharmaceutical and cosmetic industry [161]. A study conducted by Mishra et al. [162] concluded that wild edible plants play a significant role in providing livelihood and income for indigenous communities, with 5–24% (Rs 3559 to Rs 12,710) of household annual income derived from them. They also partially fulfill micronutrient requirements, yet a large deficit remains, as reflected in the low intake of wild edible plants relative to total consumption. A survey conducted by Thakur et al. [163] revealed that 50 wild species were used individually by locals, primarily in chutneys (condiments) and local brews, in the Dhauladhar region of the western Himalayan state of Himachal Pradesh. The changing lifestyle pattern was reported as the primary reason for the decline in the use of wild edible plants, as people prefer to sell them for income rather than consume them themselves, further underscoring the need for research on their sustainable utilization.

### 10.1. Bioactive Potential of Underutilized Wild Edible Berries

Numerous epidemiological studies have shown that consuming fruits and vegetables is linked to a lower risk of NCDs due to the protective effects of various antioxidants and phytochemicals that neutralise ROS, which are responsible for their onset [164]. Wild food plants are gaining attention in the food industry due to their potential to replace synthetic chemicals and nutraceuticals. Indigenous fruit crops not only exhibit adaptability to diverse environmental conditions but also possess significant nutritional and medicinal properties, highlighting their value for food security and commercialisation [165,166,167]. India is home to various wild edible plant species, such as *Rubus ellipticus* Sm, *Myrica esculenta*, *Ficus palmata*, *Berberis asiatica*, *Pyracantha crenulate*, *Morus alba* L, and *Duchesnea indica*, which are prominently and readily available in the north-western Himalayan area and have been used for many centuries for medicinal and commercial use [158]. Given the immense potential of polyphenols, extensive research is underway to improve their utilisation and oral bioavailability. A review by Zhang et al. [168] summarised various protein-, polysaccharide-, and lipid-based nanodelivery systems currently under development and research, highlighting their importance in modern health and fitness scenarios. Polyphenolic nutraceuticals currently available in the Indian market are given in Table 3.

#### 10.1.1. *Morus* Species (*M. nigra* L. and *M. alba* L.)

Mulberry is considered a highly productive woody plant known for its adaptability, ease of cultivation, and richness in bioactive compounds such as polysaccharides, alkaloids, and flavonoids, making it an ideal species for sustainable development. In the genus *Morus*, 100 subspecies have been identified, most of which are found in Asia [169]. Species plants of the genus *Morus* are mainly found in temperate and mid-elevation tropical forests, and have developed broad ecological adaptability, capable of growing in a wide variety of soils, except for swampy, saline, or highly acidic soils [170,171]. The three main species cultivated are *M. rubra* L., *M. nigra*, and *M. alba* L., due to their applicability in animal husbandry as livestock fodder and to support the silkworm industry [172]. The fruits of the *Morus* species are typically 2–3 cm in length [173]. Sugar being the primary compound responsible for sweetness in fruits, with fructose and glucose being the dominant during the fruit repining stage, in total *M. nigra* carbohydrate ranges from 5 to 19 g per 100 g of fresh fruits whereas amino acids such as glycine, aspartate and glutamate are present in the fruits with glutamate being predominantly present covering 20% of the total amino acids content [174]. The fruits of *M. nigra* are found to contain kaempferol-3-rutinoside, quercetin-3-rutinoside, and quercetin-3-glucoside, as well as anthocyanins such as cyanidin-3-rutinoside, cyanidin-3-glucoside, cyanidin-3-sophoroside, pelargonidin-3-rutinoside, and pelargonidin-3-glucoside, as well as carotenoids such as lutein, zeaxanthin, and α- and β-carotenes [175].

*Morus* species have been studied for their pharmacological activities, including anti-hypolipidemic, antidiabetic, antiobesity, antitumour, hepatoprotective, antioxidant, and neuroprotective effects, suggesting them as potential candidates for the food and healthcare industries [176].

The toxicity of various *Morus* species extracts has been evaluated, suggesting a range of 300–2000 mg/kg body weight is safe for consumption and nutraceutical and functional food use [177]. Currently mulberry species is being processed for consumption in the forms of jam, wine, yogurt, vinegar, juice, jelly, fried mulberry fruit, milk, syrup and food colorant in the industry as well as in pharmaceutical industry by incorporating them in bio-materials for treatment against various diseases such as anticancer, antioxidant and antimicrobial but challenges remains in the bioavailability of the active compounds from the fruits as well as their preservation for longer usage [178].

#### 10.1.2. *Berberis asiatica*

The genus *Berberis* is globally distributed and comprises nearly 500 species, many of which are widely cultivated for their medicinal and ornamental properties. Members of this genus are highly adaptable to adverse climatic conditions, such as tolerance to shade and drought, and the ability to thrive in open areas, forests, and wetlands [179]. In India, approximately 77 species of *Berberis* have been reported, predominantly in the Himalayan region at elevations of 1000–3000 m asl. The growing conditions around the roots of the species are reported to be soil rich in organic carbon, nitrogen, and phosphorus [180]. *B. asiatica* is the most extensive species in the western Himalayas, occurring in states including Uttarakhand, Himachal Pradesh, Jammu and Kashmir, Arunachal Pradesh, and Assam [181]. Isoquinoline alkaloids constitute the principal bioactive compounds in the genus Berberis, most prominent among them being berberine, a yellow-coloured compound identified by a tetracyclic structure composed of four fused benzene rings, with a nitrogen atom bridging two ring pairs and oxygen substitution at both ends [182]. As for the other metabolites, physicochemical evaluation revealed 1.73% tannins, 2.56% total ash, 16.44% starch, and 11.83% alcohol-soluble extractives, including phenolic compounds, flavonoids, and anthocyanins, in the plant’s roots. Moreover, fruits contain a significant amount of anthocyanin (24.59 mg/100 g of fw) [120]. Researchers have isolated the prominent compound from its fruits, berberine, along with other polyphenols, to examine its effects on various pharmacological activities, including anti-inflammatory, anti-arthritic, and protective renal effects [183,184,185]. Other parts of the species from the genus, such as roots, bark, leaves, and stems, have shown cardioprotective, neuroprotective, antimicrobial, hepatoprotective, and anticancer activity [186,187,188].

#### 10.1.3. *Duchesnea indica*

*Duchesnea indica*, also known as *Fragaria indica* Andr and *Potentilla indica*, is a perennial herb in the family Rosaceae, commonly called false strawberry, Indian strawberry, or wild strawberry. The native habitat of this species includes mountain slopes (below 3100 m asl), meadows, and riverbanks, and it especially thrives in moist, nitrogen-rich habitats [186]. It has been traditionally used for its leaves as decoration and externally for pain and swelling. The flowers are used in infusions [187]. The herb has been studied for various phenolic and phytochemical compounds, including salicylic acid (161.29 µg/g), ferulic acid (42.09 µg/g), vanillic acid (33.23 µg/g), trans-cinnamic acid (27.92 µg/g), and 4-coumaric acid (27.84 µg/g) in the ethyl extract of leaves and stems of *D. indica* [188]. Other studies have also reported several classes of phenolic compounds in the species, grouped into flavonols, ellagitannins, ellagic acid and its derivatives, hydroxybenzoic acids, and hydroxycinnamic acids [120]. Certain Chinese medical texts have reported using the aerial parts of *P. indica* for activity against cancer agents, both alone and in polyherbal formulations. Extensive studies have been conducted on *Potentilla* species regarding the abundance of their secondary metabolites, which have been shown to possess anti-inflammatory, antimicrobial, and antioxidant properties [189].

#### 10.1.4. *Ficus palmata*

The genus *Ficus* is a large group of flowering plants distributed across the Himalayan moist tropical and subtropical regions at an elevation of 1100–1800 m asl, in clay-loam soils [190,191]. It serves as an important source of fruits, vegetables, and beverages, contributing significantly to dietary diversity and local livelihoods. The medicinal potential of the genus *Ficus* has been explored over the years through ethnobotanical and pharmacological investigations, revealing its activities, including antioxidant, antimicrobial, anticancer, anti-inflammatory, and antidiabetic [192]. The hydroalcoholic extract of fruits showed analgesic activity in rats, while molecular docking suggested a potential mechanism involving COX enzyme inhibition or mu-opioid receptor (MOR) modulation, as demonstrated with diclofenac, psoralen, and rutin [193]. The phytochemical screening of *F. plamata*, which indicated the presence of various bioactive compounds, including ascorbic acid (7.27 mg/g fw), anthocyanin content (19.27 mg/100 g fw), and phenolic compounds (5.88 mg/100 g), making it a promising candidate for functional food and nutraceutical use [194,195]. The toxicity and safe human consumption were tested on rats with 70% hydroethanolic extract, and it was established at or over 2000 mg/kg body weight [193]. The fruit is currently used to make jam, jellies, and squash, and locals cook it as a vegetable. It is also utilized in the pharmaceutical industry to encapsulate bioactive compounds from the species through nanotechnology [196,197].

#### 10.1.5. *Lycium barbarum*

The *Lycium* genus has established itself as an important source of medicine and nutrition in Southeast Asia, comprising 100 species spread across temperate and tropical regions at elevations of 3063–3196 m asl. Native to arid regions, the plant grows well in well-drained, nutrient-deficient soils and exhibits remarkable resistance to drought and saline conditions [198]. The most common species of this genus found in India are *L. barbarum* L. and *L. ruthenicum* Murr., often sold as Goji berry or Wolfberry [199]. A review by Liu et al. [200] summarized the major bioactive compounds found in goji fruits, namely carotenoids, ascorbic acid, tocopherols, syringic acid, chlorogenic acid, gallic acid, caffeic acid, p-coumaric acid, 4-hydroxybenzoic acid, ferulic acid, trans-cinnamic acid, rutin, naringin, quercetin, catechin, and kaempferol, making it a superfood. Since goji berries have a high water content, they are highly perishable, limiting their use. An evaluation of the functional properties of goji berry juice demonstrated that the juice retained total phenolics of 194.5 mg gallic acid equivalent (GAE)/100 mL, flavonoids of 70.3 mg catechin equivalents (CE)/100 mL, and a total carotenoid content of 289.5 µg/100 mL. Among minerals, potassium (285.30 µg/100 mL) was the most abundant, followed by sulfur (22.30 µg/100 mL), sodium (9.37 µg/100 mL), magnesium (9.10 µg/100 mL), and calcium (8.48 µg/100 mL), demonstrating its sustainable and full utilization as a functional food [201]. Researchers investigated the incorporation of goji berry pulp into ciabatta bread, demonstrating the retention of significant amounts of bioactive compounds and notable antioxidant activity compared with the control, indicating its potential as a new functional food [202]. Currently, this species is processed into fruit juices, wines, vinegars, and yogurt. Furthermore, extracts of *L. barbarum* L. fruits have demonstrated a wide range of pharmacological activities, including anti-inflammatory, anti-aging, anti-tumor, anti-osteoporosis, anti-diabetic, heart-protective, hepatoprotective, and prebiotic activities [203,204].

#### 10.1.6. *Myrica esculenta*

*Myrica esculenta*, a perennial species well adapted to harsh environments, is commonly known as Himalayan bayberry and has regional names such as kaiphal, kaphal, maruta, and kefang. The plant is typically found in soil that is depleted in nitrogen, receiving 205–250 cm of rainfall, usually in mixed forest and forest edges [205]. Fruits are locally processed to prepare jams, syrups, and juices, thereby providing financial and nutritional security. The species is widely distributed in the hilly regions of Garhwal and Kumaon in Uttarakhand, as well as parts of Punjab and the Khasi hills of India, at an elevation of 900–2100 m above sea level [206]. The mineral and nutritional composition of *M. esculenta* fruit is documented to be 0.83% to 3.575 reducing sugars, 2.18% to 7.68% total sugars, and an overall moisture content of 72.38%. In comparison, the pulp was reported to contain 7.83% fibre, 9.62% protein, 4.93% crude fat, 78.03% carbohydrates, and an energy value of 395.04 kcal/100 g [149]. The bioactive constituents in *M. esculenta* fruit include anthocyanins (310.00 mg/100 g), carotenoids (1.43 mg/100 g), vitamin C (60.68 mg/100 g), and vitamin E (23.27 mg/100 g) [207]. Among the various biological activities, different parts of *M. esculenta* have been studied for their properties, including antiallergic and anti-allergenic effects from ethanolic bark extract, antioxidant, antipyretic, and analgesic activities from methanolic fruit extract, anti-inflammatory activity from essential oils obtained from bark, nephroprotective activity from fruit juices, as well as antiulcer, anthelmintic activity from bark, and antidiabetic effects from methanolic leaf extract. These findings highlight its significance as a potent source of nutraceuticals and functional foods [208].

#### 10.1.7. *Pyracantha crenulata*

An indigenous plant of northern and northwestern Pakistan, India, and China, *Pyracantha crenulate*, locally known as ghingaru, is commonly used in herbal medicine and traditional therapeutic preparations. In India, it is found in wild habitats across several districts of Uttarakhand, including Nainital, Almora, Pithoragarh, Champawat, Bageshwar, and Ranikhet [147]. The plant is usually found in pine and oak forests of the central Himalayas on partly shaded mountain slopes at an elevation of 1000–2400 m asl, with a preference for well-drained soil with moderate moisture [209,210,211]. This species is rich in diverse health-beneficial phytochemicals, including phenols, flavonoids, glycosides, tannins, triterpenes, alkaloids, sterols, and coumarins, with total phenolic content and flavonoid content reported as 6.59 mg/g gallic acid equivalent and 7.46 mg/g quercetin equivalent, respectively [147,212]. Another review by Song et al. [159] documented the presence of health-beneficial compounds, including gallic acid (10.15 mg/100 g fw), catechin (8.27 mg/100 g fw), anthocyanins (0.44 mg/100 g fw), vitamin C (3.30 mg/100 g fw), β-carotene (5.08 μg/g of extract), and condensed tannins, suggesting the importance of this plant against various metabolic diseases.

Researchers investigating this wild plant have revealed antioxidant, antiproliferative, antiurolithogenic, anti-inflammatory, and anticancer activities across various formulations, particularly using ecologically friendly nanoparticles. These formulations have successfully reduced the number of cancer cells in Swiss albino mice, underscoring their importance as potent candidates in both the food and pharmaceutical industries [213].

#### 10.1.8. *Rubus ellipticus*

*Rubus ellipticus*, also known as the golden Himalayan raspberry and locally as “hisalu,” is a plant with various medicinal properties, from traditional use as a root poultice to treat bone fractures and fever [160]. This species is native to India and Sri Lanka in tropical and subtropical regions, mostly in mountains and lowlands, and is available in wild habitats [161]. The plant has shown rapid growth in open, sunny areas as well as in dense rainforests, occurring at elevations ranging from 300 to 2600 m above sea level, with annual rainfall of 2000 to 6000 mm [154]. The fruit is rich in various nutrients and bioactive compounds, including 4.73% protein, 2.35% fibre, 0.96% fat, 86.4% carbohydrates, and an ash content of 2.97 g/100 g of dry matter [154]. Mineral content of the species is reported to be 89.43 mg sodium, 1.82 mg potassium, 0.95 mg calcium, 118.72 mg magnesium, 0.020 mg copper, 12.77 mg zinc, and 4.249 mg iron/100 g of dry weight (dw), and 19.8 mg ascorbic acid/100 g fw [154]. The polyphenolic compounds that have been reported in the fruits include rutin, quercetin, quercetin 3-O-glucuronide, epicatechin, phlorizin, epigallocatechin, cyanidin, chrysin, pelargonidin, malic acid, gallic acid, ellagic acid, citric acid, chlorogenic acid, and some amino acids like tyrosine, hydroxyproline, serine, histidine, and leucine [154]. Due to the diverse metabolites present in various parts and extracts of the plant, they have been employed to test their activity against inflammation, malaria, diabetes, cancer, nephrodegeneration, and neurodegeneration [214]. Currently, the fruits of the species are used to make jams, jellies, and desserts, and are known to have very little economic value, indicating a lack of awareness of the plant’s protection and sustainable use for pharmaceutical and nutraceutical purposes, despite its antioxidant richness [215].

## 11. Challenges in Harnessing and Utilising Wild Fruits to Their Full Potential

The limited shelf life of these wild fruits remains the main obstacle; thus, it is essential to develop and implement value-addition and environmentally friendly strategies to utilise these fruits beyond their fresh consumption. Integration of traditional practices with modern scientific approaches represents a promising avenue for enhancing healthcare outcomes, as traditional knowledge can serve as an important foundation for the discovery and development of novel drugs and dietary supplements [206]. Researchers have continually explored greener methods of extracting beneficial bioactive compounds from plant material. Among the many factors that determine extraction yield, optimal extraction conditions remain the one for which there are few standardized techniques for different target compounds. It is therefore essential to identify the optimal and economically viable conditions [216]. The color, stability, and expression of these bioactive compounds after extraction are highly influenced by many factors, such as pH, solvent type, temperature, concentration, molecular structure, oxygen exposure, light, enzymatic activity, and storage and processing conditions. These factors can also lead to the degradation of these compounds. A review by Narra et al. [217] discussed various thermal processing techniques like boiling, steaming, superheated steaming, blanching, and microwaving, and their effect on phenolic compounds, carotenoids, glucosinolates (GLs), and ascorbic acid present in fruits and vegetables, as it can affect the nutraceuticals’ bioavailability in the body. The paper highlighted that, since polyphenols are prone to heat-mediated degradation, the optimization of an appropriate extraction method remains a gap. The author’s findings, based on total phenolics, total flavonoids, and antioxidant assays, revealed that superheated steaming retained the highest levels of bioactive compounds, followed by microwave heating. Hence, new strategies are being researched to fully utilize the bioactive compounds from these fruits, such as microencapsulation. A study reported that locals benefit annually from *Myrica esculenta* fruits, selling them for 1.4 million Indian rupees per season. However, no data are available on the commercialisation of other wild fruits, which opens the way for their valorisation and for self-employment opportunities for the local Himalayan communities [218].

## 12. Conclusions and Future Perspective

Wild fruits from the Himalayan belt are a promising source of nutraceuticals and functional foods for managing early symptoms of endometriosis, given their rich nutrient and bioactive compound profiles. This review examines biomarkers involved in the progression and symptoms of endometriosis. It discusses how phenolic compounds, primarily found in these wild fruits, mediate these processes. The review also analyzes the prominent wild fruits available in Uttarakhand, their in vitro and in vivo pharmacological activities, traditional uses, and current commercialization. The review also highlights current challenges that hinder the full utilization of bioactive compounds from these wild fruits in the food and pharmaceutical industries, thereby paving the way for further research. A lack of standardized extraction methods and poor storage techniques for these wild fruits still pose challenges. Future research should focus on developing nutraceuticals and functional foods that encapsulate bioactive compounds without significant degradation, using environmentally friendly and cost-effective techniques.

## Figures and Tables

**Table 1:** Phenolic and non-polyphenolic bioactive compounds in the management of endometriosis-related symptoms evaluated using in vitro and in vivo experimental systems.

Compound	Experimental System	Molecular Mechanism of Action	Significant Results	Reference
Quercetin and kaemferol	Luciferase-expressing FVB female mice and primary human endometrial stromal cells	Modulation of NR4A1-mediated cellular pathways	Decreased Luciferase activity of ectopic lesions at 100 mg/kg/day. Inhibited mTOR. Decreased fibrosis progression.	[125]
Quercitin	Female SD rats	Modulation in the HPGA axis	Decrease in the level of FSH and LH. Downregulation of ER*α* and PR in the endometrium.	[126]
	HEC-1-A cells	Inducing ferroptosis in HEC-1-A cells	Inhibition of proliferation at 104.2 μM at 24 h	[127]
Resveratrol	Female SD rats	Migitating the activation of PPARγ (Peroxisome proliferator-activated receptor gamma)	Decreased lesion size, alteration in macrophage polarisation, glucose tolerance, and adipocyte size	[128]
	Endometriotic stromal cells	Activation of Sirtuin 1	Suppressed expression of IL-8 in endometriotic stromal cells.	[129]
	Human endometrial stromal cells	Increase in the expression of the antiapoptotic BCL2 gene	Pterostilbene inhibited the proliferation of cells. Resveratrol and its analogs increased the number of apoptotic cells.	[130]
Gallic acid	Female C57BL/6 mice and Human Endometrial Epithelial Cells.	Downregulation of cyclin D1 expression of PI3K/AKT pathway genes	Gallic acid inhibited endometrial epithelial cell proliferation. Alleviates endometrial hyperplasia	[131]
Caffeic acid	Ectopic endometrial cells	Increase in Nrf-2 gene expression, indicating a possible modulation via Nrf-2/HO-1/NQO1 pathway	Downregulated ROS levels, HO-1 and NQO1 activity.	[132]
Berberine	AN3 CA and HEC-1-A Endometrial cancer cells	Modulation in miR-101/COX-2 axis	Repressed migration and inhibited invasion potential of cells in cell lines	[133]
Berberine and Carvacol	Female Kunming mice	Inhibition of NF-κB and MAPK pathway activation	Downregulated the mRNA expression of TLR2 and TLR4. Inhibition of phosphorylation of NF-κB and MAPK signaling pathway proteins. Downregulation of TNF-α, IL-1β, IL-6, and IL-8 expression.	[134]
Oleuropein	C57BL/6J, luciferase-expressing FVB, FVB, and SCID female mice and HeLa cells	Suppression of the TNFα-induced phosphorylation of Akt and p44/p42 MAP kinase	Selective inhibition of Erβ activity in HeLa cells at 10 nM. Suppressed progression of ectopic lesions in SCID mice. Upregulated the levels of the cleaved form of caspase 3, reactivating apoptosis in epithelial and stromal cells of ectopic lesions.	[135]
Protoberberine (coptisine, berberine, sanguinarine, and stylopine)	Female Wistar rats	Upregulation of the concentration of metabolites involved in energy homeostasis, including glucose, lactate, and glutamate	Ectopic lesions were decreased.	[136]

Abbreviations: AKT: Protein Kinase B, BCL2: B-cell lymphoma 2, FSH: Follicle-stimulating hormone, HEC-1-A: Human endometrial adenocarcinoma cell line, HPGA: Hypothalamic–Pituitary–Gonadalaxis, HO-1: Heme oxygenase-1, IL-8: Interleukin-8. LH: Luteinizing hormone, MAPK: Mitogen-Activated Protein Kinase, mTOR: mechanistic target of rapamycin, NQO1: NAD(P)H: quinone oxidoreductase 1, NR4A1: Nuclear receptor subfamily 4 group A member 1, Nrf-2: Nuclear factor erythroid 2-related factor 2, PI3K: Phosphoinositide 3-kinase, PPARγ: Peroxisome Proliferator-Activated Receptor Gamma, TLR: Toll-like receptor.

**Table 2:** Summary of traditional uses and bioactivity identified in wild Himalayan fruits.

Fruits	Common Name	Traditional Uses	Bioactive Compounds Identified	Parts	References
*Myrica esculenta*	Kaphal, kaiphal, katphal, and kataphal	Jaundice, fever, bronchitis, dysentery, body ache, headache, ulcer	Proanthocyanins, alkaloids, tannins, glycosides, gallic acid, hexadecanol, ascorbic acids, cis-β-caryophyllene, n-octadecanol, phytosterols, saponins, catechin, chlorogenic acid, trans-cinnamic acid, p-coumaric acid, hydroxybenzoic acid, gallic acid, caffeic acid, methyl salicylate, O-amino benzohydroxamic acid, pyridine, and ellagic acid.	Stem, leaves, and fruit	[149,150]
*Morus nigra* and *Morus alba*	Shahtoot	Prevention of various diseases of the liver, kidney, and aging	Tannins, coumarins, triterpene, anthocyanins (cyanidin and delphinidin), catechin, chlorogenic acid, and 3-hydroxybenzoic acid, 4-hydroxybenzoic acid, vanillic acid, rutin, quercetin-3-O-glucoside, ascorbic acid, resveratrol, β-carotene, gallic acid, and fatty acid (palmitic acid, linolic acid, and oleic acid)**.**	Leaves, fruits	[151,152,153]
*Rubus ellipticus*	Hisaul, hisalu	Mature fruits are used in the treatment of coughs and fever, and roots and shoots are well known for their renal tonic, anti-diuretic, and diarrhoea, dysentery	β-carotene, anthocyanins (cyanidin and delphinidin), ascorbic acid, rutin, caffeic acid, gallic acid, m-coumaric acid, chlorogenic acid, 3-hydroxybenzoic acid, 4-hydroxybenzoic acid, ferulic acid, ellagic acid, phloridzin, and vanillic acid.	Leaves, roots, shoots, and fruit	[152,154,155]
*Berberis asiatica*	Kingor, Chutro, rasanjan (Nep); marpyashi (Newa); daruharidra, darbi (Sans)	Jaundice, diabetes mellitus, wound healing, asthma; drying unhealthy ulcers, anti-inflammatory, swelling, treating pneumococcal infections, eye (conjunctivitis) and ear diseases, rheumatism, fever, stomach disorders, skin disease (hyperpigmentation), malarial fever	Carotenoids (α and β-carotene), ascorbic acid, anthocyanins, gallic acid, catechin, caffeic acid, chlorogenic acid, and coumaric acid	Roots, shoot, stem, bark, and fruit	[152,156]
*Potentilla indica*	False strawberry	Leprosy, tissue inflammation, congenital fever, cancer, and diabetes mellitus	Sterols, volatile oils, ellagitannins, ellagic acid and its derivatives, hydroxybenzoic acid, and hydroxycinnamic acid, brevifolin carboxylate, caffeic acid, acarbose, ascorbic acid, 2,4-dichloro-6-hydroxy-3,5-dimethoxytoluene, and 2-methyl-6-(4-methylphenyl)-2-hepten-4-one.	Whole plant	[120,157]
*Pyracantha crenulata*	Ghigharu	Cardioprotective, anti-hypertensive, antioxidants, reduction in cholesterol, anti-malarial	Ascorbic acid, β-carotene, lycopene, catechin, anthocyanins (cyanidin and delphinidin), gallic acid, phloridzin, ferulic acid, chlorogenic acid, 3-hydroxybenzoic acid, 4-hydroxybenzoic acid, caffeic acid, *m*-coumaric acid, *p*-coumaric acid, ellagic acid, and condensed tannins	Stem, bark, leaf	[152,158,159]

**Table 3:** Current polyphenolic nutraceuticals present in the market.

Product	Ingredients	Dosage	Target	Manufactured by
HealthyHey grape seed extract	Grape seed extract	500 mg	Antioxidant activity	HealthyHey Foods LLP, Mumbai, India
Green tea leaf extract	Green tea leaf extract, *Piper nigrum* extract	650 mg	Anti-inflammatory, anti-aging, weight-mediating	MyFitFuel, Inventiva Labs Pvt Ltd., Delhi, India
Now Foods, natural resveratrol	*Polygonum cuspidatum* extract complex, grape seed extract	200 mg	Cardiovascular support, anti-ageing, anti-inflammatory	Now Foods, USA
Quercetin with Bromelain health capsules	Quercetin, bromelain	1200 mg	Antioxidant, anti-inflammatory	SAS India PVT LTD
Resveratrol complex	Japanese knotweed extract, grape seeds, red wine, blueberry, grape skin	1800 mg	Antioxidant	Piping Rock Health Products, USA
Polyphenol complex	A blend of pomegranate, citrus bioflavonoids, grape seed, resveratrol, apple peel, blueberry, cranberry, strawberry, acerola, acai, turmeric, broccoli seed, green tea, quercetin, olive leaf, and ginkgo leaf extracts.	2250 mg	Antioxidant	UBNA distribution LLC, USA
Apple polyphenols	Apple extract standardised to 80% polyphenols and 5% phloridzin	600 mg	Antioxidant, cardioprotective, weight and cholesterol lowering, and oral health management,	Super smart, USA

## Data Availability

No new data were created or analyzed in this study. Data sharing is not applicable to this article.
